# Management of Diabetic Kidney Disease With Persistent Hypotension: A Case of Lifestyle-Driven Renal Recovery

**DOI:** 10.7759/cureus.93484

**Published:** 2025-09-29

**Authors:** Sungmin Song, Okoro Obioha, Yoobin Kang

**Affiliations:** 1 Medicine, Trinity School of Medicine, Warner Robins, USA; 2 Pediatric Medicine, Central Georgia Pediatrics, Macon, USA; 3 School of Medicine, Trinity School of Medicine, Warner Robins, USA

**Keywords:** chronic kidney disease (ckd), complication of type-2 diabetes mellitus, hypotension treatment, types 2 diabetes, underdeveloped community

## Abstract

Diabetic kidney disease (DKD) is typically accompanied by hypertension, which enables guideline-directed renoprotective therapy. We describe a 61-year-old woman with type 2 diabetes mellitus (T2DM) and persistent hypotension (≈92/55-98/65 mmHg) without albuminuria, in whom angiotensin-converting enzyme (ACE) inhibitors/angiotensin receptor blockers (ARBs) and sodium-glucose cotransporter-2 (SGLT2) inhibitors were deemed unsafe due to baseline low blood pressure. Management, therefore, emphasized non-pharmacologic measures, tight glycemic control, weight reduction (~10 lb), hydration, and avoidance of nephrotoxins in a resource-limited setting. Renal function improved from an eGFR of 55 mL/min/1.73 m² (2023) to 69 (2024) and stabilized at 67.2 (2025) with HbA1c 6.3-6.6% and continued lack of albuminuria; neurologic and ophthalmic diabetic manifestations remained stable. This case illustrates a guideline gap: when persistent hypotension and absent albuminuria preclude standard agents, individualized metabolic and lifestyle strategies may achieve renal stabilization. Although causality cannot be inferred, the temporal association between improved weight/glycemia and estimated glomerular filtration rate (eGFR) recovery suggests a pragmatic pathway for hypotensive T2DM patients outside conventional algorithms.

## Introduction

Diabetic kidney disease (DKD) is the leading global cause of chronic kidney disease and end-stage kidney disease, contributing substantially to cardiovascular morbidity and mortality [1], and patients with DKD experience an elevated risk of premature death compared with diabetes alone [2]. Hypertension is the predominant hemodynamic phenotype in DKD, and the presence of albuminuria and/or reduced eGFR identifies individuals at greater severity and risk of progression [3], whereas a non-albuminuric phenotype is increasingly recognized and has been associated with adverse outcomes despite the absence of proteinuria [4]. Guidelines emphasize renin-angiotensin system blockade with ACE inhibitors or ARBs once albuminuria is present or eGFR falls below 60 mL/min/1.73 m² [5], yet controversies in chronic kidney disease (CKD) blood pressure management persist, particularly when hypotension complicates therapeutic decision-making [6]. The large hypertension guidelines further caution that aggressive pharmacologic lowering may be unsafe in patients with already low baseline blood pressure [7]. From a pathophysiologic standpoint, diabetes-related autonomic neuropathy with impaired baroreflexes can contribute to chronic hypotension, narrowing the therapeutic window for renoprotective agents [8]. In such cases, especially when albuminuria is absent, individualized care emphasizing glycemic optimization, lifestyle measures, and nephrotoxin avoidance becomes central, as highlighted in the present case of a woman with DKD and chronic hypotension without albuminuria who could not receive standard agents yet experienced renal improvement from stage 3 to stage 2, illustrating a real-world gap in current recommendations.

## Case presentation

A 61-year-old Asian woman, a never-smoker, who denies alcohol use, had long-standing T2DM. Family history included maternal hypotension and paternal T2DM with laryngeal cancer. She lived in a rural industrial area with limited medical oversight and long-standing misconceptions that low blood pressure (BP) was protective, contributing to poor adherence. At baseline in 2016, renal function was preserved with an eGFR of 63 mL/min/1.73m² and normal serum creatinine. Glycated hemoglobin (HbA1c) was 7.5%, and serum electrolytes (sodium, potassium, chloride, calcium, and bicarbonate) were consistently within normal limits, thereby excluding secondary metabolic or endocrine causes of hypotension. In particular, the absence of electrolyte derangements ruled out adrenal insufficiency, thyroid dysfunction, and renal tubular acidosis as contributors. The chronic course of low blood pressure, absence of systemic symptoms, and positive maternal history of lifelong hypotension suggested a constitutional predisposition. Furthermore, in the context of long-standing diabetes, autonomic dysfunction with impaired baroreflexes was considered a likely mechanism, accounting for the patient’s persistent low BP despite otherwise stable metabolic status (Table 1).

**Table 1:** Laboratory trends (2016–2025). Albuminuria assessed by dipstick/ACR; “negative” indicates ACR <30 mg/g or negative dipstick. eGFR: Estimated glomerular filtration rate, BMI: Body mass index

Year	HbA1c (%)	Fasting Glucose (mg/dL)	eGFR (mL/min/1.73m²)	Creatinine	Albuminuria	BMI/Weight	Notes
2016	7.5	262	63.2	Normal	Negative	27.8	Dx T2DM, poor adherence
2018	7.8	271	61.5	Normal	Negative	28.2	Chronic cough
2020	7.6	265	60.3	Normal	Negative	28.3	Poor adherence
2022	7.4	244	60.1	Normal	Negative	27.7	Neuropathy onset
2023	7.2	254	55.78	Normal	Negative	27.4	Gastritis, weight loss
2024	6.5	144	69.1	Normal	Negative	24.4	Improved control
2025	6.3	120	67.39	Normal	Negative	24.2	Stable

T2DM was recognized in 2016 (glucose >250 mg/dL). Metformin was prescribed at that time, but adherence was poor and limited to episodes of severe hyperglycemia. Baseline eGFR was 63.2 mL/min/1.73m² with elevated HbA1c (7.6); BP averaged approximately 90/60 mmHg. From 2018 to 2021, she presented sporadically with hyperglycemia. Investigations for chronic cough were unrevealing, and treatment adherence remained poor.

**Figure 1:**
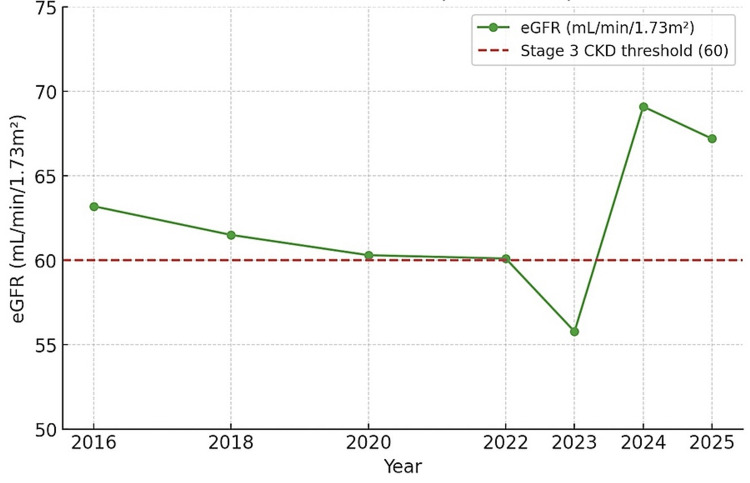
Longitudinal eGFR values abstracted from clinic records (2016–2025). The image is created by the author. eGFR: Estimated glomerular filtration rate

In 2022, she developed peripheral neuropathic symptoms, including facial tingling and distal sensory changes, which improved over three months with better glycemic control. By 2023, she developed diabetic gastritis, presenting with poor appetite and weight loss. Renal function declined to eGFR 55.7 mL/min/1.73m² (stage 3 CKD). At that point, she was counseled on disease progression and began taking metformin consistently for the first time (Figure 1). Given persistent hypotension and absence of albuminuria, angiotensin-converting enzyme inhibitor (ACEi) or ARB therapy was not initiated. Management, therefore, focused on glycemic control and lifestyle measures.

In early 2024, her diabetic gastritis worsened, further reducing food intake and indirectly improving glycemic control through sustained weight loss. Endoscopic evaluation confirmed chronic gastritis without malignancy. Omeprazole was initiated at that time, leading to symptomatic improvement by mid-2024. Laboratory evaluation revealed renal recovery: eGFR 69 mL/min/1.73m² (stage 2 CKD), HbA1c 6.5%, normal creatinine, and absent albuminuria. BP remained between 88/55 and 95/65 mmHg (Table 2).

**Table 2:** Summary of outpatient blood pressure recordings (2016–2025). Values represent ranges across 2–3 visits per year. SBP: Systolic blood pressure, CKD: Chronic kidney disease, DBP: Diastolic blood pressure

Year	Avg SBP (mmHg)	Avg DBP (mmHg)	Notes
2016	99	67	Initial diagnosis
2018	97	65	Poor adherence
2020	98	66	CKD borderline
2022	96	64	Neuropathy onset
2023	95	64	Stage 3 CKD; gastritis, weight loss
2024	95	67	Persistently hypotensive
2025	96	66	Stable

By mid-2025, she remained clinically stable with sustained metformin use, omeprazole therapy, and dietary adherence. At her most recent follow-up in June 2025, her weight was 64 kg (body mass index [BMI], approximately 24.7). She continued to report persistent polyuria without incontinence, distal sensory decrease without progression, and preserved vision despite vitreous floaters. Laboratory evaluation demonstrated stable renal function with eGFR 67 mL/min/1.73m², serum creatinine 0.91 mg/dL, and blood urea nitrogen (BUN) 12 mg/dL. Electrolytes were normal (sodium 143 mEq/L, potassium 4.2 mEq/L, chloride 106 mEq/L, calcium 9.8 mg/dL), as were liver function tests (aspartate aminotransferase (AST) 23 U/L, alanine aminotransferase (ALT) 16 U/L, albumin 4.4 g/dL). HbA1c was 6.3% with fasting glucose 120 mg/dL, while the lipid panel showed low-density lipoprotein (LDL) 62 mg/dL, high-density lipoprotein (HDL) 57 mg/dL, and triglycerides 97 mg/dL. Urinalysis remained negative for proteinuria or hematuria. Collectively, these findings confirmed stable stage 2 CKD with preserved electrolyte balance despite persistent hypotension (Table 3).

**Table 3:** Most recent laboratory data (June 2025). HbA1c: glycated hemoglobin test, eGFR (MDRD): Estimated glomerular filtration rate, BUN: Blood urea nitrogen, Na: Sodium, K: Potassium, Cl: chloride, AST/ALT: Aspartate aminotransferase/Alanine aminotransferase, CBC: Complete blood count

Test	Result	Reference range	Interpretation
HbA1c	6.30%	4.8 – 5.9 %	Mildly elevated
Glucose (fasting)	120 mg/dL	70 – 100 mg/dL	Elevated
eGFR (MDRD)	67.39 mL/min/1.73m²	>60	CKD stage 2
BUN	12 mg/dL	8 – 20 mg/dL	Normal
Creatinine	0.91 mg/dL	0.6 – 1.2 mg/dL	Normal
Na	143 mEq/L	135 – 147	Normal
K	4.2 mEq/L	3.5 – 5.1	Normal
Cl	106 mEq/L	95 – 110	Normal
Calcium	9.8 mg/dL	8.5 – 10.5	Normal
Albumin	4.4 g/dL	3.5 – 5.5	Normal
AST/ALT	23/16 IU/L	<40	Normal
Total bilirubin	0.2 mg/dL	0.3 – 1.2	Slightly low
Lipids	LDL 62 / HDL 57 / TG 97	LDL goal <100	Well-controlled
CBC	Hb 12.6 g/dL / WBC 4.4 x10⁶/µL / Plt 223	Within normal limits	Normal
Urinalysis: Negative for protein, glucose, blood; SG 1.005 (dilute but no proteinuria).			

Yearly averages in Tables 1-3 and Figures 1 were derived from 2-3 outpatient clinic visits per year, with values abstracted directly from the patient’s chart. These have been summarized into aggregate tables for clarity. Original clinic notes are available to the editorial office upon request.

## Discussion

Clinical dilemma

Persistent hypotension in T2DM is rarely described, whereas hypertension predominates. Most patients with T2DM and CKD develop hypertension, and ACEi or ARB therapy is recommended, particularly at eGFR <60 or with albuminuria. In this patient, T2DM was first recognized in 2016 with glucose >250 mg/dL. In 2022, she developed peripheral neuropathic symptoms, which improved with better glycemic control. By 2023, she had developed diabetic gastritis with weight loss. Her renal function declined to an eGFR of 55 mL/min/1.73m² in 2023, ordinarily warranting ACEi or ARB therapy, but chronic hypotension contraindicated their use, and the absence of albuminuria further reduced the rationale. Management, therefore, focused on intensive metabolic control and lifestyle modification, leading to partial renal recovery (eGFR 69 in 2024 and 67 in 2025), indicating the potential for renal stabilization through non-pharmacologic measures when guideline-directed therapy cannot be applied.

Possible mechanisms

Several mechanisms may explain this patient’s persistent hypotension. From the clinical data, serum electrolytes (sodium, potassium, chloride, calcium, and bicarbonate) remained consistently within normal limits, effectively excluding metabolic or endocrine causes such as adrenal insufficiency, thyroid dysfunction, and renal tubular acidosis. The chronic, asymptomatic low BP course and maternal history of lifelong hypotension further suggest a constitutional predisposition. In the context of long-standing diabetes, autonomic dysfunction with impaired baroreflexes is a plausible mechanism, supported by evidence that diabetic autonomic neuropathy can disrupt cardiovascular autonomic regulation and cause hypotension [9]. Cardiac autonomic neuropathy (CAN), a subset of diabetic autonomic neuropathy, is well described in the literature and may manifest as orthostatic hypotension, resting low BP, and impaired heart rate variability [10].

Therapeutic considerations

Sodium-glucose cotransporter-2 (SGLT2) inhibitors are a cornerstone in diabetic CKD and reduce kidney disease progression [11,12]. However, they also lower BP modestly by ~2-4 mmHg, which may be undesirable in baseline hypotension [13]. Glucagon-like peptide-1 (GLP-1) receptor agonists reduce major cardiovascular events [14] and, in meta-analyses, improve cardiovascular mortality and renal outcomes [15], with clinically meaningful glycemic and weight benefits that appear pronounced in Asian populations. Although not used in this case, GLP-1 receptor agonists remain a reasonable future option, with clinically meaningful glycemic and weight benefits that appear especially pronounced in Asian populations [16].

Management and literature context

In this patient, non-pharmacologic strategies were not merely supportive but appeared central to renal recovery. Sustained weight loss (~10 lb), strict dietary adherence, and consistent glycemic control coincided with an improvement in eGFR from 55 to 69 mL/min/1.73m², which then stabilized over two years. While causality cannot be definitively proven, recent studies suggest that lifestyle interventions, such as weight reduction, dietary optimization, and improved glycemic management, can attenuate CKD progression and, in select cases, improve renal indices independent of pharmacotherapy. This underscores the significance of lifestyle-driven management as a pragmatic alternative when guideline-directed renoprotective agents are contraindicated, particularly in hypotensive, non-albuminuric DKD phenotypes [17].

## Conclusions

In summary, this case highlights the management challenges of T2DM complicated by CKD and persistent hypotension. At an eGFR of 55 mL/min/1.73m² in 2023, ACEi or ARB therapy would usually be indicated, but chronic hypotension and absent albuminuria made renoprotective pharmacotherapy unsafe. With lifestyle modification, strict glycemic control, and patient education, renal function improved to stage 2 CKD and remained stable through 2025. The case underscores the need for individualized care and clearer guidance for hypotensive diabetic CKD phenotypes. This case further highlights the correlation between glycemic control, as measured by HbA1c, and kidney function, as measured by eGFR, even when BP is not elevated.
